# An Integrated Electronic Patient-Reported Outcome-Guided COPD Management Strategy Incorporating Adjunctive Acupuncture: Study Protocol for a Randomized Controlled Trial

**DOI:** 10.3390/healthcare14203509

**Published:** 2026-10-19

**Authors:** Yangjia Wu, Zuoqin Yang, Chan Xiong, Xuyan Yang, Guang Chen, Fuyu Peng, Yilin Liu, Fanrong Liang, Yuting Wang

**Affiliations:** 1 Acupuncture and Tuina School, Chengdu University of Traditional Chinese Medicine, No. 37, Shierqiao Road, Chengdu 610075, China; wyj599@stu.cdutcm.edu.cn (Y.W.); tcm_chen7410@163.com (G.C.); pengfuyu@stu.cdutcm.edu.cn (F.P.); liuyilin980515@163.com (Y.L.); 2 Key Laboratory of Acupuncture for Senile Disease, Chengdu University of Traditional Chinese Medicine, Ministry of Education, Chengdu 610075, China; 3 The Department of Acupuncture, No. 3 Affiliated Hospital of Chengdu University of Traditional Chinese Medicine (West District), Chengdu 611730, China; yzq@cdutcm.edu.cn; 4 The Department of Respiratory Medicine, No. 3 Affiliated Hospital of Chengdu University of Traditional Chinese Medicine (West District), Chengdu 611730, China; xiongchan@cdutcm.edu.cn; 5 The Department of Geriatrics, No. 3 Affiliated Hospital of Chengdu University of Traditional Chinese Medicine (West District), Chengdu 611730, China; yangxuyan.1997@outlook.com

**Keywords:** COPD management, adjunctive acupuncture, electronic patient-reported outcome, hospitalization, healthcare

## Abstract

**Background/Objectives:** Chronic obstructive pulmonary disease (COPD) is characterized by persistent symptoms and fluctuating disease status. Effective COPD management requires not only alleviating symptom burden but also maintaining disease stability. An integrated management strategy added to usual care, combining adjunctive acupuncture for symptom control with electronic patient-reported outcome (ePRO)-guided symptom monitoring and timely clinical responses, may provide a novel approach to optimize COPD care. This trial will evaluate whether this management strategy can improve long-term COPD outcomes compared with usual care alone. **Methods:** This is an open-label, randomized controlled trial with blinded outcome assessments. A total of 172 patients with stable COPD will be randomized 1:1 to usual care or intervention group. The intervention consists of an initial symptom-control phase followed by an ePRO-guided monitoring and response phase with predefined clinical management adjustments. We will follow up participants for 12 months and collect data every 2 months to assess hospitalization, exacerbations, mortality, health status and safety. All analyses will adhere to the intention-to-treat principle. The primary outcome (i.e., time to first COPD-related hospitalization) will be analyzed using a cause-specific Cox proportional hazards model, with death before hospitalization treated as a competing event. The cumulative incidence of COPD-related hospitalization will be estimated using the Aalen–Johansen estimator. **Conclusions:** This study will evaluate, compared with usual care alone, whether an integrated ePRO-guided COPD management strategy incorporating adjunctive acupuncture could provide additional benefits. As this is the first trial of this integrated intervention, the findings should be considered preliminary and cannot determine the separate effects of the individual components. This study may provide a patient-centered management model for COPD and inform future exploration of integrative care.

## 1. Introduction

Chronic obstructive pulmonary disease (COPD) is a common respiratory disease characterized by persistent airflow limitation and progressive respiratory symptoms. It is one of the leading causes of death worldwide [1]. Despite guideline-based treatment, many patients with COPD continue to experience substantial symptom burden and remain at risk of exacerbations and hospitalization [2]. Common symptoms include dyspnea, chronic cough (sometimes with phlegm) and reduced exercise tolerance. COPD symptoms may fluctuate over time and often worsen before a clinically recognized exacerbation occurs. Therefore, effective management of COPD should aim not only to alleviate current symptom burden but also to maintain long-term disease stability [3,4].

To address persistent symptom burden, adjunctive interventions may complement usual COPD care. Among these interventions, acupuncture has been investigated as a potential option for symptom relief in COPD. In a placebo-controlled randomized trial, when added to usual care, acupuncture reduced respiratory symptoms, particularly dyspnea on exertion, and improved health-related quality of life [5]. Subsequent trials and meta-analyses have reported consistent findings [5,6,7,8,9]. These findings suggest that acupuncture may serve as an adjunctive component within COPD management to provide additional symptom improvement.

Nevertheless, symptom improvement represents only one aspect of effective COPD management. Maintaining long-term disease stability is another goal for COPD management [10], which is usually supported by scheduled clinical visits for monitoring symptom changes and guiding ongoing disease management [2]. However, symptom fluctuations between visits may be difficult to capture. Electronic patient-reported outcome (ePRO) systems may help address this limitation through remote and continuous monitoring, which has been increasingly incorporated into chronic disease management [11,12,13]. Patient-reported outcomes (PROs) can capture changes in COPD symptom status that are perceived as important by patients over time [14]. Studies have shown that worsening PRO scores were associated with symptom deterioration and exacerbations in patients with COPD [15,16], suggesting that systematic monitoring of PROs may help identify patients who require additional clinical attention. Furthermore, evidence from a trial that used ePRO for symptom monitoring, with alerts to support management in patients with multiple chronic diseases (including COPD), suggested potential benefits for reducing hospitalization [17]. Symptom monitoring via ePRO may represent a promising approach to complement COPD management for maintaining disease stability [18].

Taken together, we designed an integrated ePRO-guided COPD management strategy incorporating adjunctive acupuncture as part of long-term COPD care. The intervention consists of an initial symptom-control phase followed by an ePRO-guided monitoring and response phase. This initial treatment phase will use adjunctive acupuncture alongside usual care to support symptom control before participants enter the longitudinal ePRO monitoring phase, during which symptom trajectories will be tracked and ePRO monitoring will inform timely adjustment of COPD care. We aimed to evaluate whether this multicomponent management strategy could provide additional clinical benefits compared with usual care alone.

### Objective

This trial aims to evaluate the preliminarily effects of an integrated management strategy, compared with usual care alone, on COPD-related hospitalization and other clinical outcomes in patients with COPD.

## 2. Methods

### 2.1. Study Design

This trial is a multicenter, randomized, controlled, parallel-group superiority trial with open-label treatment and blinded endpoint assessments. A total of 172 participants will be randomized 1:1 to receive usual care alone or usual care plus an integrated ePRO-guided COPD management strategy incorporating adjunctive acupuncture. We will follow up participants for 12 months to assess whether the ePRO-guided COPD management strategy, compared with usual care alone, can reduce COPD-related hospitalization, mortality and improve health status. The assessments will be conducted at baseline and every 2 months after randomization.

We designed this study in accordance with the Declaration of Helsinki and have registered the trial with the International Traditional Medicine Clinical Trial Registry (register number: ITMCTR2026001109, https://itmctr.ccebtcm.org.cn/mgt/project/view/2049067921937924096, register date: 28 April 2026). We followed the Standard Protocol Items: Recommendations for Interventional Trials (SPIRIT 2025) guidelines to develop this protocol (version 1.0, 21 January 2026) [19]. Table 1 provides the detailed trial schedule.

**Table 1:** Trial schedule.

Procedure/Assessment	Screening			Trial Management					
	V0			V1	V2	V3	V4	V5	V6
	Screening	Randomization	Initial Treatment *	Month 2	Month 4	Month 6	Month 8	Month 10	Month 12
Enrollment screening ^1^	X								
Informed consent	X								
Baseline assessment ^2^	X								
Randomization		X							
Intervention ^3^		X	X *	X	X	X	X	X	X
Usual care		X	X *	X	X	X	X	X	X
Pulmonary function test	X								X
Borg Dyspnea Scale after 6MWT ^4^	X					X			X
Self-reported COPD exacerbations ^5^				X	X	X	X	X	X
COPD-related hospitalization ^6^				X	X	X	X	X	X
Healthcare utilization ^7^				X	X	X	X	X	X
Mortality				X	X	X	X	X	X
SGRQ ^8^	X					X			X
Scheduled clinical visit				X	X	X	X	X	X
CCQ monitoring ^&^			X ^9^	X ^10^	X ^10^	X ^10^	X ^10^	X ^10^	X ^10^
ePRO monitoring pathway ^11^				X ^11^	X ^11^	X ^11^	X ^11^	X ^11^	X ^11^
Intervention implementation record ^12^				X ^12^	X ^12^	X ^12^	X ^12^	X ^12^	X ^12^
Safety assessment			X ^13^	X	X	X	X	X	X

Note: 6MWT, 6 min walk test; COPD, chronic obstructive pulmonary disease; SGRQ, St George’s Respiratory Questionnaire. CCQ, Clinical COPD Questionnaire. NA, not applicable. * The initial treatment refers to the initial one-month course of adjunctive acupuncture (i.e., 12 sessions) treatment. Only the participants allocated to the intervention group will receive the initial treatment. ^&^ CCQ is the ePRO monitoring tool. Only the participants allocated to the intervention group will undergo ePRO monitoring. 1. Enrollment screening: see details of the eligibility criteria. During screening, prior COPD-related hospitalization, emergency visits, treated exacerbations, and routine respiratory-clinic consultations will be distinguished and verified from available medical records. 2. Baseline assessments include demographics, blood pressure, heart rate, medical history, medication history, physical examination, and pregnancy test (if applicable). 3. The intervention group will receive the integrated management strategy. 4. The Borg Dyspnea Scale (0–10; higher scores indicate greater dyspnea) will be administered after the 6 min walk test. 5. The new onset or worsening of one or more respiratory symptoms (cough, sputum, wheezing, dyspnea, or chest tightness) lasting at least 2 days. 6. COPD-related hospitalization: a hospital admission primarily attributable to acute exacerbation of COPD (with or without pneumonia). 7. Healthcare utilization refers to any emergency department visits and hospitalization verified by the hospital records. 8. SGRQ (0–100) evaluates health-related quality of life, with higher scores indicating worse quality of life. 9. The CCQ score will be assessed at the end of the initial acupuncture course (i.e., after completion of the 12 sessions). 10. The participants in the intervention group will complete weekly CCQ report via ePRO platform during the 12-month follow-up (Supplementary File S1). 11. The participants in the intervention group will undergo ePRO monitoring, which may trigger automated alerts, unscheduled clinical visits and repeat acupuncture courses (Supplementary File S1, Figure 1). 12. The participants in the intervention group will document the intervention implementation records including ePRO completion rate, number of alerts, clinical contact measures, repeat acupuncture exposure and others (Supplementary File S1). 13. The participants in the intervention group will also receive safety assessment during the initial acupuncture treatment.

**Figure 1:**
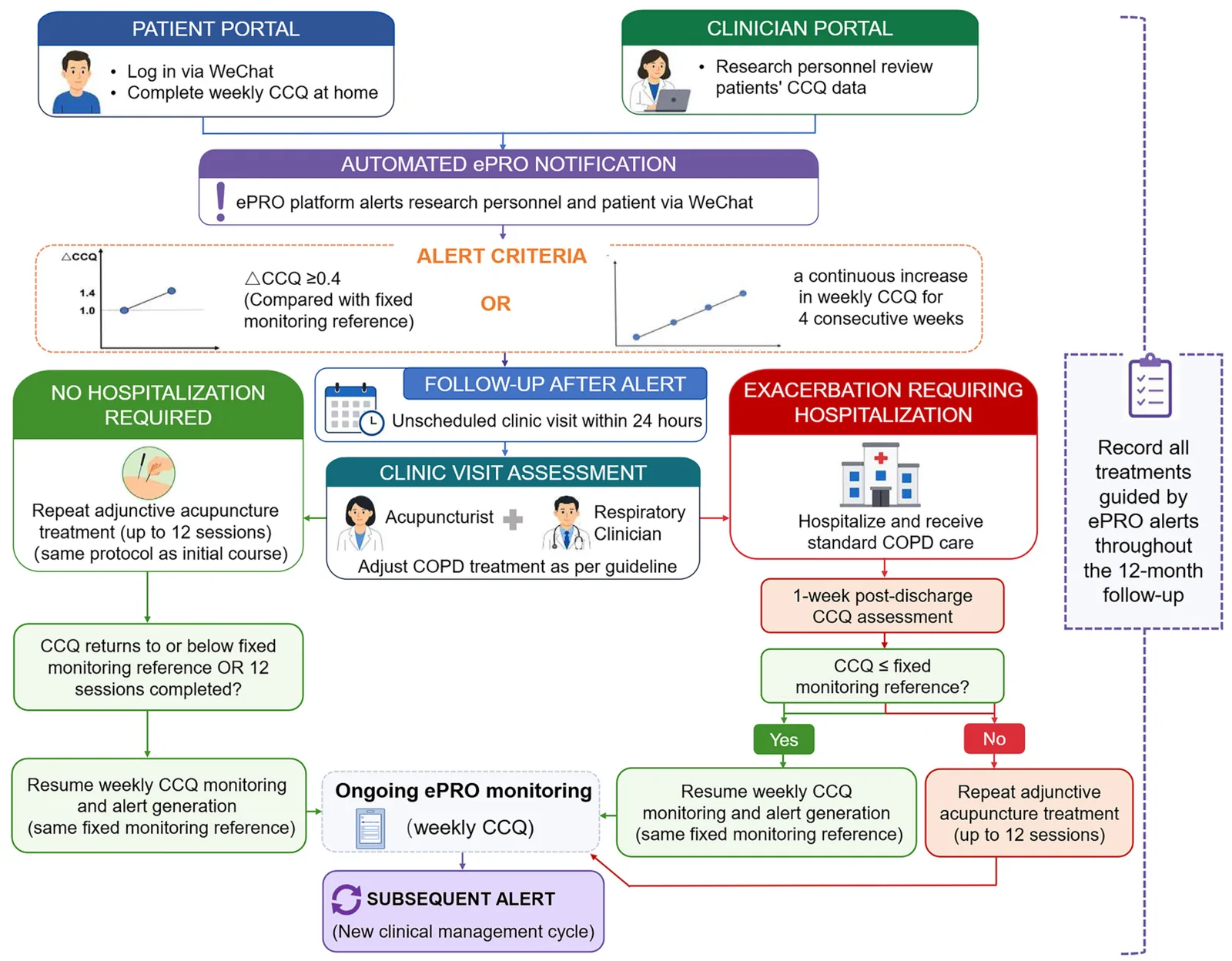
The ePRO alert workflow. Note: 1. The ePRO platform, accessible via WeChat for both patients and clinicians, supports weekly Clinical COPD Questionnaire (CCQ) reporting and monitoring (Supplementary File S1). 2. Following completion of the initial acupuncture treatment, participants will enter the longitudinal ePRO monitoring phase (Supplementary File S1). 3. An automated alert is triggered when the CCQ score exceeds the fixed monitoring reference obtained after the initial acupuncture treatment by a minimal important difference (MID) of 0.4 or shows a monotonic increase over four consecutive weeks.

### 2.2. Trial Setting

Participants will be recruited from outpatient clinics at the participating centers. Potentially eligible patients will be identified during routine clinical visits by clinicians and trained investigators. All assessments will be conducted online and at outpatient clinics. The acupuncture intervention will be performed in the department of acupuncture following a standardized protocol.

### 2.3. Eligibility Criteria

Patients meeting all the following criteria will be further evaluated for eligibility: (1) age 40–80 years. (2) COPD confirmed according to the Global Initiative for Chronic Obstructive Lung Disease (GOLD) criteria with a post-bronchodilator FEV_1_/FVC ratio < 0.70 at screening. Acceptable spirometry requires at least three acceptable maneuvers (i.e., the best two values of FEV_1_ and FVC should be within 5% or 150 mL of each other). (3) Patients with Stage II, III, or IV COPD based on the Global Initiative for Chronic Obstructive Lung Disease (GOLD) criteria and with a forced expiratory volume in one second (FEV_1_) ≥ 25% and <80% of the predicted value. (4) No acute exacerbation and no sign of edema within 4 weeks prior to enrollment. (5) Receiving stable treatment according to GOLD recommendations for at least 3 months prior to enrollment, with no changes in the treatment regimen. (6) Able to walk independently and perform activities of daily living. (7) At least one hospital visit or consultation at a respiratory clinic for COPD within the past 12 months. (8) Informed consent obtained from patients or their legal representatives.

We will exclude patients with one of the following exclusion criteria: (1) patients with language, memory, cognitive or psychiatric disorders, or with limited education (i.e., below junior high school) that may prevent them from understanding the questionnaires or operating electronic tools; (2) patients with the following comorbidities: chronic heart failure, autoimmune diseases, chronic renal failure, chronic hepatic failure, or malignant tumors; (3) patients with the following respiratory diseases: active pulmonary tuberculosis, bronchial asthma, severe bronchiectasis, or interstitial lung disease; (4) history of lung surgery for COPD, including segmentectomy, wedge resection, lobectomy, pneumonectomy, or lung volume reduction surgery (including bronchoscopic lung volume reduction); (5) patients requiring long-term oxygen therapy (LTOT) (oxygen therapy > 15 h/day); (6) current participation in another clinical trial or having participated in another clinical trial within 90 days preceding trial inclusion; (7) history of any acupuncture treatment in the prior 1 month; (8) unable to undergo acupuncture due to local skin infection at or near the acupoints, limb defects, or other conditions; (9) women who are pregnant or in lactation.

### 2.4. Randomization and Blinding

**Figure 2:**
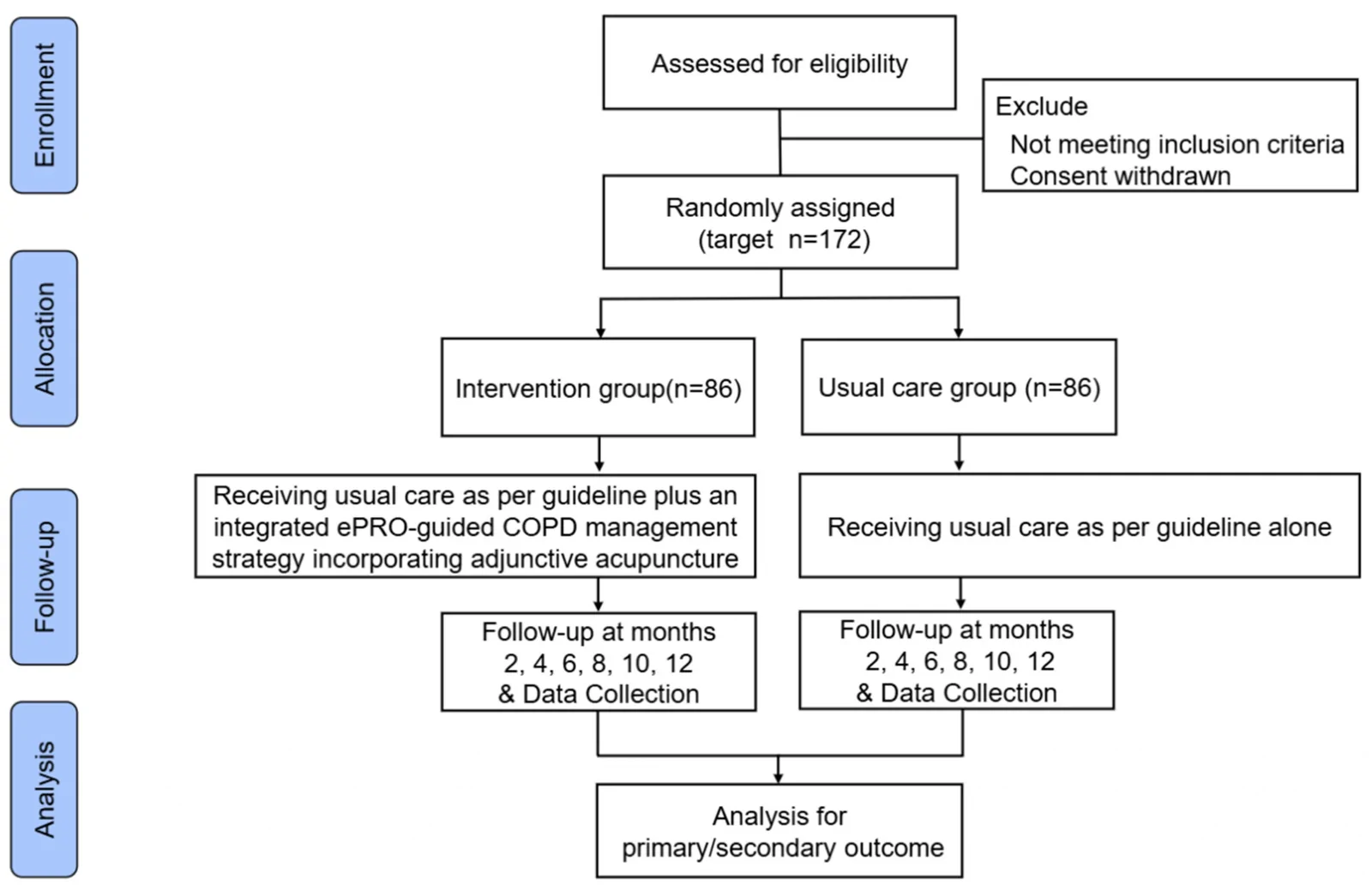
Study flow diagram. Note: ePRO, electronic patient-reported outcome.

We will randomize patients via an Interactive Web Randomization System (IWRS) maintained by the coordinating center at Chengdu University of Traditional Chinese Medicine. An independent statistician will generate the randomization sequence using R. Eligible patients will be randomly assigned in a 1:1 ratio to the control or intervention group using center-stratified block randomization with randomly varying block sizes (2, 4, or 6). This is an open-label trial in which only the outcome assessors, data collectors, outcome adjudicators and statisticians will remain blinded (Supplementary File S1). Figure 2 shows the study flowchart.

### 2.5. Interventions

#### 2.5.1. Control Group: Usual Care

After randomization, all participants will receive a standardized one-page COPD education leaflet, which includes written instructions to seek urgent care for acute deterioration. Patients in both groups will attend scheduled clinical visits every 2 months and receive usual care by the clinicians who are advised to manage their patients according to published guidelines [20]. At a minimum, usual care will include regular and long-term maintenance of COPD pharmacotherapy, inhaler technique education, smoking cessation support, and treatment adjustment or escalation as per guideline. If participants experience an acute exacerbation, they will receive rescue medication, emergency care, or hospitalization as determined by the clinicians. All relevant healthcare utilization and treatment details will be documented.

#### 2.5.2. Intervention Group: An Integrated Management Strategy

In addition to usual care, participants allocated to the intervention group will receive an integrated management strategy, which consists of an initial symptom-control phase followed by an ePRO-guided monitoring and response phase. The details are described below.

Participants will receive an initial one-month course of adjunctive acupuncture (i.e., 12 sessions) as the first component of the management strategy. This initial treatment phase is intended to provide additional symptom control alongside guideline-based COPD treatment before the longitudinal ePRO monitoring phase begins [9]. The acupoints were selected based on our previous study, including bilateral Feishu (BL13), Pishu (BL20), Shenshu (BL23), Zusanli (ST36), Zhongfu (LU1), Fenglong (ST40), and Dingchuan (EX-B1) (Supplementary File S2) [9].

Following completion of the initial symptom-control phase, participants will enter the longitudinal ePRO monitoring phase (Supplementary File S1). ePRO monitoring is used to identify symptom deterioration during follow-up and support timely management, including the repetition of adjunctive acupuncture.

The ePRO platform will monitor the symptom trajectories, which includes a patient portal and a clinician portal. Patients provide their data by logging into the patient portal via WeChat (Figure 1). Research personnel can monitor patients’ data via the clinician portal. Participants will complete the Clinical COPD Questionnaire (CCQ) weekly at home. The CCQ is a validated patient-reported instrument that assesses COPD symptoms, functional status, and mental state. Previous studies have shown that worsening CCQ scores were associated with increased risk of COPD exacerbations [21]. It was therefore selected as the symptom-monitoring PRO tool in this study.

The CCQ score obtained at the end of the initial acupuncture course (i.e., after the completion of the 12 sessions) will serve as the fixed monitoring reference for subsequent symptom monitoring, which will not be reset during follow-up. No other CCQ assessments will be performed during the initial acupuncture treatment period after randomization.

The ePRO platform will automatically notify both the clinicians and patients when predefined alert criteria indicating symptom deterioration are met. The criteria include either 1) a pragmatic threshold of a 0.4-point increase (i.e., the minimal important difference [MID]) [21,22] in the CCQ total score compared with the monitoring reference; or 2) if the increase does not reach 0.4 points, a strictly monotonic increase in CCQ total score (i.e., CCQ_1_ < CCQ_2_ < CCQ_3_ < CCQ_4_) across four consecutive weeks (Figure 1).

Following each alert, research personnel will coordinate an unscheduled clinic visit at which a respiratory clinician and an acupuncturist will assess the participant’s condition within 24 h. The clinician will determine appropriate treatment adjustments as per guidelines [2]. If hospitalization is not indicated, adjunctive acupuncture treatment will be repeated, with up to 12 sessions (Supplementary Files S1 and S2). Patients presenting with an exacerbation requiring hospitalization will not receive acupuncture, but repeat acupuncture may be administered after clinical recovery (Supplementary Files S1 and S2, Figure 1). We will record all treatments following the ePRO alerts (Figure 1). Supplementary File S1 provides more details.

#### 2.5.3. Cointervention and Intervention Implementation Records

During the trial, any cointerventions, including contaminant acupuncture and medication changes, will be recorded throughout the trial for both arms. In addition, we will document the intervention implementation records to characterize intervention delivery for each participant in the intervention group. The records include ePRO completion rate, number of alerts, clinical contact measures, repeat acupuncture exposure and others (Supplementary File S1).

### 2.6. Outcome Assessment

#### 2.6.1. Outcome Measures

Blinded outcome assessors will contact patients every 2 months for 12 months to assess the clinical outcomes and collect information on the details of treatment and healthcare utilization (Table 1). Outcome measures include:

• COPD-related hospitalization: a hospital admission primarily attributable to acute exacerbation of COPD (with or without pneumonia).
• Self-reported COPD exacerbations: defined as the new onset or worsening of one or more respiratory symptoms (cough, sputum production, wheezing, dyspnea, or chest tightness) lasting for at least two consecutive days, with moderate and severe events defined by the need for oral corticosteroids and/or antibiotics and emergency department visits or hospitalization, respectively, and episodes separated by at least seven symptom-free days.
• Healthcare utilization: including any emergency department visits and hospitalization verified by the hospital records.
• Mortality: including COPD-related mortality and all-cause mortality.
• Health-related quality of life will be assessed using St. George’s Respiratory Questionnaire (SGRQ) (ranging from 0 to 100), with higher scores indicating poorer health-related quality of life.
• Dyspnea on exertion will be assessed using the Borg Dyspnea Scale immediately after the 6 min walk test (6MWT).
• Pulmonary function will be assessed by spirometry, with FEV_1_ used as the primary pulmonary function indicator.

#### 2.6.2. COPD-Related Hospitalization Criteria and Outcome Adjudication

All participating centers will use prespecified criteria to assess hospitalization in patients with suspected acute exacerbation of COPD. Hospitalization will be considered for patients with any of the following criteria: (1) presence of severe or rapidly worsening respiratory symptoms; (2) insufficient response to appropriate outpatient management; (3) clinically significant hypoxemia or worsening respiratory failure; or (4) a need for hospital-based treatment or monitoring that cannot be safely provided in the outpatient setting. All hospitalization events will be documented and verified using hospital records and independently adjudicated by two blinded adjudicators, with disagreements resolved by consensus or, when necessary, by a third independent adjudicator. COPD-related hospitalization will be defined and classified according to the prespecified adjudication criteria detailed in Supplementary File S1.

#### 2.6.3. Primary Outcome

The primary outcome is the time from randomization to first COPD-related hospitalization. COPD-related hospitalization refers to a hospital admission primarily attributable to acute exacerbation of COPD (with or without pneumonia).

#### 2.6.4. Secondary Outcome

The secondary outcomes include: (1) the number of self-reported COPD exacerbations over 12 months; (2) the number of COPD-related hospitalization or emergency department visits over 12 months; (3) COPD-related mortality at 12 months; (4) all-cause mortality at 12 months; (5) change in dyspnea on exertion (the Borg scale score after the 6 min walk test) from baseline to 12 months; (6) change in the score of St. George’s Respiratory Questionnaire (SGRQ) from baseline to 12 months; (7) change in FEV_1_ as a percentage of predicted value from baseline to 12 months.

### 2.7. Sample Size

The time to first COPD-related hospitalization will be the primary outcome. The primary analysis will follow the intention-to-treat principle, using a Cox proportional hazards model to compare hospitalization risk between the intervention and usual care groups. The sample size was therefore calculated based on the log-rank test under the proportional hazards assumption, assuming all the included participants would finish the 12-month follow-up. Based on data from comparable populations in China, we assumed a 1-year cumulative incidence of COPD-related hospitalization of 40% in the usual care group [23,24,25]. Because no previous studies have evaluated a similar strategy, we assumed the treatment effect as a 20% absolute reduction informed by COPD management trials reporting hospitalization outcomes [26,27]. Therefore, with a two-sided α of 0.05, 80% power, 1:1 allocation, and anticipated hazard ratio (HR) of 0.44, about 46 primary events would be required, resulting in a sample size of 154 participants. Allowing for 10% loss to follow-up, the final sample size was 172 participants (86 per group) (R software, version 4.5.0, R Foundation for Statistical Computing, Vienna, Austria). Given that this is the first trial to evaluate the integrated management strategy and the evidence to inform the expected hospitalization risk and treatment effect is limited, this trial is intended to be exploratory.

### 2.8. Safety Monitoring

The steering committee (YW, FL, ZY) and the ethics committees will monitor the conduct and safety of the trial. All adverse events (AEs) will be continuously monitored and recorded throughout the study to evaluate the safety of the interventions. Details about the procedures for dealing with AEs are provided in Supplementary File S1.

### 2.9. Data Management

All deidentified data will be recorded in case report forms (CRFs) and then entered into a secure, web-based electronic data capture (EDC) system (Supplementary File S1). All documents will be securely stored in locked cabinets. Research personnel will receive training in advance, and data management staff will regularly monitor data quality and integrity. Access will be limited to the principal investigator and authorized study personnel. The data will be used solely for research purposes.

### 2.10. Quality Control and Adherence

Before trial implementation, all investigators, clinicians, acupuncturists and research personnel will receive standardized training. All study procedures will be conducted according to the predefined protocol. The implementation of the acupuncture intervention, ePRO-guided management pathway, follow-up assessments, and protocol deviations will be documented throughout the trial.

To enhance long-term follow-up adherence, the ePRO platform will provide automated reminders for scheduled assessments, and study coordinators will contact participants when assessments are missed or incomplete (Supplementary File S1). Similar strategies will be applied to acupuncture adherence, with participants receiving reminders for scheduled sessions and follow-up contact after missed appointments to identify barriers and facilitate rescheduling when appropriate. Participant adherence, including ePRO completion and acupuncture session attendance, will be recorded and evaluated throughout the trial. Participants who discontinue or deviate from the allocated intervention will, whenever possible, remain in follow-up, and all available primary and secondary outcome data will continue to be collected according to the study schedule.

Regular monitoring will be conducted to ensure data quality and trial compliance. Research personnel will review study records, identify protocol deviations, and verify the completeness and accuracy of collected data.

### 2.11. Statistical Analysis

Analyses will follow the intention-to-treat (ITT) principle [28]. The statistical inference will be based on the two-sided 95% confidence interval and corresponding *p*-values. All analyses will be performed using R statistical software. Baseline characteristics between groups will be described using means and standard deviations (SDs) or medians and interquartile ranges (IQRs) for continuous variables, and frequencies and percentages for categorical variables.

The treatment effects estimated in the analyses will reflect the effect of the integrated strategy rather than the individual components. We defined the primary estimand as the cause-specific HR for COPD-related hospitalization comparing the intervention group with the control group over 12 months, with death before hospitalization treated as a competing event. The primary analysis will use a cause-specific Cox proportional hazards regression model, adjusted for center, presenting results as HR and their 95% confidence intervals (CIs). As a sensitivity analysis, the Cox model will be further adjusted for baseline covariates (i.e., age, baseline FEV_1_% predicted, history of acute exacerbations/prior hospitalization, smoking status and coronary artery disease). We will apply the Aalen–Johansen cumulative incidence function estimator to calculate the cumulative incidence of the COPD-related hospitalization over 12 months [29]. The proportional hazards assumption will be assessed by log–log survival plots and a treatment-by-time interaction term in the Cox model; non-parallel plots and a large, statistically significant interaction will indicate non-proportionality [30]. If the assumption is violated, the cause-specific Cox model will remain the primary analysis, with the HR interpreted cautiously; 12-month cumulative incidence risk ratios and risk differences estimated using the Aalen–Johansen estimator will be reported as complementary measures. Sensitivity analyses will be performed using Fine–Gray regression to estimate the sub-distribution HR for COPD-related hospitalization, which will be interpreted as a complementary measure of the treatment effect [29].

All secondary effect analyses will be considered exploratory. Repeated counts of exacerbation episodes and COPD-related hospitalization or emergency department visits over time will be compared between groups using negative binomial regression to account for overdispersion, with results presented as rate ratios and 95% CIs [31]. The log-transformed person-time at risk, calculated from randomization until death, loss to follow-up or study completion, will be included as an offset term to account for differences in actual observation duration. We will use modified Poisson regression (12-month mortality) to estimate the risk ratio with a sandwich robust variance for 95% CI calculation. For Borg dyspnea and SGRQ scores, baseline-adjusted repeated-measures ANCOVA models will be used, with the 6- and 12-month assessments included. For FEV_1_% predicted, assessed at baseline and 12 months, a standard baseline-adjusted ANCOVA model will be used. ANCOVA models will estimate the mean differences between groups and the 95% CIs.

Missing baseline covariates and missing continuous outcomes due to loss of follow-up will be handled using multiple imputation by chained equations (MICE) under the missing-at-random (MAR) assumption. To impute death-related missingness, we will use ‘composite-outcome’ strategy with ‘while-alive’ strategy as the sensitivity analysis [32]. For participants who died, we will assign a score of 10 to the Borg Dyspnea Scale (i.e., the maximal breathlessness), 100 to SGRQ (i.e., worst possible health status), and 0 to FEV1% predicted (i.e., the worst possible function). Details of the missing-data handling for continuous outcomes are provided in Supplementary File S1. In addition, we will directly report the number of adverse events in each group. The trial results will be reported following the Consolidated Standards of Reporting Trials (CONSORT) guidelines [33].

### 2.12. Patient and Public Involvement

Patients were not involved in the design of this study. During the trial, patients will report their symptoms through the ePRO platform, and these data will be used for monitoring.

## 3. Discussion

This trial will provide evidence on whether an integrated management strategy, delivered in addition to guideline-based usual care, provides additional clinical benefit for patients. The results will reflect the overall effect of the integrated management strategy, without distinguishing the effects of individual components. The proposed strategy is designed to complement routine COPD care and address two major challenges in COPD management—persistent symptom burden and fluctuating disease status—by integrating adjunctive acupuncture with ePRO-guided monitoring and timely clinical responses. Given the exploratory nature of the trial, the findings should be interpreted as preliminary evidence and used to inform future confirmatory trials.

To address different aspects of disease management across the continuum of care, COPD management typically relies on multiple complementary interventions [34,35,36]. Accordingly, previous COPD management trials have commonly adopted integrated multicomponent intervention designs rather than evaluating individual interventions in isolation [34,35,37,38]. Consistent with this approach, the present trial evaluates an integrated strategy. As with other multicomponent interventions, the relative contribution of the individual components in this study (e.g., ePRO monitoring, adjunctive acupuncture, treatment adjustment, and increased clinician–patient interaction) cannot be determined. The findings will therefore be interpreted as the overall effect of the strategy rather than the contribution of any single component.

Usual care for COPD primarily consists of guideline-recommended pharmacological therapy combined with non-pharmacological interventions such as pulmonary rehabilitation, smoking cessation support, vaccination, and patient education. These strategies form the basis of long-term COPD management [37]. Beyond usual care, a wide range of studies have explored additional COPD management strategies, including multidisciplinary disease-management programs, self-management interventions, community-based care models, and home-based rehabilitation programs [37,38,39,40]. These strategies aimed to improve long-term disease control and reduce mortality and healthcare utilization. However, the magnitude and consistency of these benefits varied across studies, particularly with regard to hospitalization and mortality outcomes [34,35].

In this context, if proven effective, the ePRO-guided COPD management strategy proposed here may have several implications for COPD management. It may provide a feasible model for delivering COPD care supported by ePRO monitoring. In healthcare systems where acupuncture is available, the findings may further inform multidisciplinary COPD care incorporating adjunctive acupuncture.

This study has several strengths. First, we will conduct the trial with rigorous methodological standards. The intervention follows a standardized, predefined management strategy, including prespecified acupuncture protocol and ePRO-guided management rules, to enhance consistency and reproducibility across study sites. The ePRO platform supports standardized longitudinal data collection and real-time monitoring throughout the 12-month follow-up, which could improve follow-up completeness and facilitate adherence. Automated reminders via ePRO platform and active follow-up procedures are expected to enhance intervention adherence and minimize missing data throughout the trial. Another strength is the comprehensive outcome assessments, with COPD-related hospitalization as an objective primary endpoint and secondary outcomes capturing mortality, quality of life, lung function, and exercise capacity from both clinical and patient-centered perspectives.

This study also has several limitations. Due to the nature of the intervention, double blinding is not feasible in this trial. Therefore, we adopted an open-label design. However, we will apply the IWRS to ensure allocation concealment, and outcome assessors and statisticians will remain blinded to minimize potential bias. Because participants were not blinded, PROs may be subject to reporting bias. The requirement for participants to be able to use electronic devices, together with the exclusion of patients with several common comorbidities, may limit the generalizability of the findings. The sample size estimation was informed by indirect evidence. Therefore, if the observed treatment effect differs from the assumed effect size, the findings should be interpreted cautiously considering the magnitude, precision, and clinical relevance of the estimated treatment effect. The study was designed to detect a 20% absolute difference in the primary outcome, but the true effect may be smaller. Because this trial compares a multicomponent management strategy with usual care, the contribution of individual components cannot be determined. In particular, the intervention group receives more frequent symptom monitoring and may have additional clinical contact, which cannot be separated from the overall intervention effect. Future studies may evaluate the individual components if the exploration becomes a research priority.

## 4. Conclusions

This study will evaluate, compared with usual care alone, whether an integrated ePRO-guided COPD management strategy incorporating adjunctive acupuncture provides additional clinical benefits. The trial is designed to assess the preliminary effect of the strategy as a whole, while the relative contribution of individual components will require further investigation. The findings may provide a patient-centered management model for COPD and inform future development of multidisciplinary care.

## Data Availability

No new data were created or analyzed in this study. Data sharing is not applicable to this article.

## Supplementary Materials

The following supporting information can be downloaded at https://www.mdpi.com/article/10.3390/healthcare14203509/s1. Supplementary File S1: Additional Methodological Details [32,41,42,43]; Supplementary File S2: Details of the Administration of Acupuncture [44,45]; Supplementary File S3: SPIRIT 2025 Checklist.
