# Supplementary material for: An Integrated Electronic Patient-Reported Outcome-Guided COPD Management Strategy Incorporating Adjunctive Acupuncture: Study Protocol for a Randomized Controlled Trial
Source: Healthcare (Basel). 2026 Oct 19;14(20):3509. doi: 10.3390/healthcare14203509 (PMC13704764; doi:10.3390/healthcare14203509)
Supplement: Supplementary file 1 [file healthcare-14-03509-s001.zip › Supplementary File S1.pdf]

Supplementary File S1. Additional Methodological Details

1. Allocation Management

This is an open-label trial; allocation concealment will be ensured by a centralized randomization system. A blinding Coordinator will serve as the interface between the centralized randomization system and the intervention team and will communicate the assigned intervention to the participant and treating clinicians and acupuncturists. Outcome assessors, data collectors, outcome adjudicators, and statisticians will not participate in the process stated above and will remain blinded to treatment allocation. Any accidental disclosure of allocation to blinded study personnel will be documented and reported to the principal investigator (YW).

2. The Detailed ePRO-guided Intervention Algorithm

2.1 ePRO Monitoring and Alert Management

2.1.1 Initiation of ePRO Monitoring

Participants allocated to the intervention group will first receive an initial course of adjunctive acupuncture consisting of 12 sessions (*Appendix 2*). Following completion of the 12-session initial acupuncture course, participants will enter the longitudinal ePRO monitoring phase. The CCQ score obtained at the end of the initial acupuncture course will serve as the fixed monitoring reference for subsequent follow-up. No other CCQ assessments will be performed during the initial acupuncture treatment period after randomization. The monitoring reference CCQ value will remain unchanged throughout the 12-month follow-up and will not be reset after an alert, clinical recovery, hospitalization, or repeat acupuncture treatment.

2.1.2 Weekly CCQ Monitoring

During the longitudinal ePRO monitoring phase, participants will complete the CCQ once weekly at home through the ePRO system accessed via WeChat. The completed CCQ assessments will be automatically transmitted to the ePRO platform, where research personnel can review participants' symptom trajectories through the clinician portal.

2.1.3 Handling of Missing CCQ Assessments

If a participant does not complete a scheduled weekly CCQ assessment, the ePRO system will issue an automated reminder on the scheduled assessment day. If the CCQ assessment remains incomplete 4 hours after the first reminder, a second automated reminder will be issued. If the assessment remains

incomplete 24 hours after the scheduled assessment time, the study personnel will contact the participant and provide additional reminders or assistance as appropriate. A missing CCQ assessment will not be interpreted as symptom deterioration and will not independently trigger an ePRO alert. If the CCQ cannot ultimately be obtained, the assessment will be recorded as missing and will not be imputed for the purpose of alert generation.

2.1.4. Handling of Technical Failures

If a participant is unable to complete a scheduled CCQ assessment because of a technical failure of the ePRO system, the event and its timing will be documented by the study personnel. When feasible, the participant will complete the CCQ using a prespecified alternative method, including the website linked to the ePRO system. If electronic completion remains unavailable, a paper-based CCQ may be used, and the completed assessment will be entered into the ePRO system by the study personnel. Technical failure will not independently trigger an alert.

2.1.5 Alert Generation

When either criterion is met, the ePRO system will automatically generate an alert and notify both the participants and the study personnel. An alert will be generated if either of the following criteria is satisfied:

- The CCQ total score increases by ≥0.4 points compared with the fixed monitoring reference;
- When the increase does not reach 0.4 points, the CCQ total score shows a strictly monotonic increase across four consecutive weekly assessments: $CCQ_1 < CCQ_2 < CCQ_3 < CCQ_4$.

2.1.6 Alert Review

The time at which the ePRO system generates an alert will be defined as T0 and will serve as the starting point for the 24-hour clinical response window. The designated member of the study team will review the alert through the clinician portal and coordinate an unscheduled clinical assessment. A two-person on-call arrangement will be established covering all days, including weekends and public holidays. The first member will serve as the primary reviewer. If the primary reviewer is unavailable, the second will serve as the backup reviewer.

2.1.7 Clinical Response after an Alert

Within 24 hours after an alert, the participant will be assessed by a respiratory clinician and an acupuncturist. The respiratory clinician will determine whether the participant has experienced clinical

deterioration or a COPD exacerbation, whether adjustment of usual COPD care is indicated according to relevant clinical guidelines and whether hospitalization is required.

1）Management Without Hospitalization

If hospitalization is not required, the respiratory clinician will adjust usual COPD treatment, as appropriate, in accordance with relevant guidelines, and a repeat course of acupuncture will be initiated. During the repeat acupuncture course, ePRO alert generation will be suspended.

Each repeat acupuncture course will comprise up to 12 sessions following the same acupuncture protocol as the initial course (*Appendix 2*). During the repeat acupuncture course, participants will continue to complete the CCQ weekly; however, ePRO alert generation will be suspended. The course will be discontinued once the participant's CCQ score returns to or below the fixed monitoring reference or when 12 sessions have been completed, whichever occurs first. If the participant's CCQ score remains above the fixed monitoring reference after 12 sessions, the course will nevertheless be considered complete. Following completion of the repeat acupuncture course, weekly CCQ monitoring and alert generation will resume using the same fixed monitoring reference. Any subsequent alert will be managed as a new clinical management cycle.

2) Management Following Hospitalization

If hospitalization is required, acupuncture will not be administered during hospitalization, and ePRO alert generation will be suspended. No CCQ assessments will be required during hospitalization. One week after discharge, the participant will complete a scheduled CCQ assessment. The resulting CCQ score will determine whether repeat acupuncture is initiated.

1) If the CCQ score has returned to or below the fixed monitoring reference, repeat acupuncture will not be initiated, and weekly CCQ monitoring and ePRO alert generation will resume using the same fixed monitoring reference. Any subsequent alert will be considered a new alert episode and managed as a new clinical management cycle.

2) If the CCQ score remains above the fixed monitoring reference, repeat acupuncture will be initiated according to the repeat acupuncture process described above. Any subsequent alert will be considered a new alert episode and managed as a new clinical management cycle.

2.1.8 Definitions of Alert Episodes

1) Duplicate Alerts

If repeated alerts are generated before the initial alert is reviewed and managed, these alerts will be considered duplicate alerts and will be treated as part of the same alert episode. They will not be counted as additional alert episodes.

2) Alert-Free Interval

The alert-free interval is defined as the period during which ePRO alert generation is suspended during repeat acupuncture or hospitalization. No new alerts will be generated during this period. Alert generation will resume when ePRO alter generation is restarted after completion of repeat acupuncture or after hospital discharge. No fixed duration will be specified for the alert-free interval, which will depend on the duration of the corresponding duration of repeat acupuncture or hospitalization.

3) Recurrent Alerts and Maximum Number of Alert Episodes

After ePRO alert generation has resumed, any subsequent alert meeting the predefined alert criteria will be considered a recurrent alert and will be managed according to the clinical response pathway described above. There will be no fixed maximum number of alert episodes during the overall follow-up period.

3. Intervention Implementation Records

Intervention implementation records will be prospectively recorded throughout the trial to characterize intervention delivery and fidelity to the prespecified management algorithm. The records include ePRO implementation measures, alert process measures, clinical contact measures, repeated acupuncture exposure and adherence, as well as the ePRO system performance records.

a. ePRO implementation measures: ePRO completion rate calculated as completed CCQ assessments / scheduled CCQ assessments × 100%;

b. Alert process measures include number of alters (i.e., each ePRO alert episode will be counted once, regardless of the number of repeated notifications) and time from alter to assessment (i.e., unscheduled clinical visit);

c. Clinical contact measures include number of clinical contacts (excluding scheduled clinical visits), contact duration per time and management decision;

d. Repeated acupuncture exposure and adherence: total number of acupuncture courses initiated following ePRO alerts, sessions per repeat course;

e. ePRO system performance records: number of false alerts, number of missed alerts and number of delayed alerts.

4. Blinded Outcome Adjudication

All hospitalization events will be documented and verified using hospital records. Each hospitalization will be independently adjudicated by two independent blinded adjudicators, with disagreements resolved by consensus; if consensus cannot be reached, a third independent adjudicator will be consulted.

Information from hospital records will be used to ascertain the exact admission and discharge dates, as well as the clinical status at discharge (recovered, unchanged, worsened and died). Based on the documentation, we will classify the primary reason for hospitalization as

a. Acute exacerbation of COPD (with or without pneumonia);

b. Cardiovascular disease;

c. Other causes.

When multiple diagnoses are documented, the first-listed diagnosis will be considered the primary reason if it is consistent with the description of chief complaint and medical history. Otherwise, consensus will be reached for the primary reason. When an acute exacerbation of COPD and pneumonia coexist, the event will be classified as a COPD-related hospitalization regardless of which diagnosis is listed first. Deaths occurring during a COPD-related hospitalization will be classified as COPD-related deaths. Deaths due to other causes or with an undetermined cause (e.g., death outside hospital) will be classified as all-cause mortality.

We will adopt the following definition to define an event of hospitalization:

1) An inpatient admission with a length of stay exceeding 24 hours;

2) Transfers between hospitals will be considered a single hospitalization event when a participant is discharged from one hospital and directly admitted to another hospital, without discharge to home;

3) Readmissions after discharge will be considered new hospitalization events;

4) The date of initial admission will be defined as the event date for each hospitalization.

5. Adverse Event and Safety Management

Adverse events (AEs) are defined as any unfavorable or unintended medical occurrence in a participant after enrollment, regardless of whether it is considered related to the study intervention. A serious adverse event (SAE) is defined as any AE that results in death, is life-threatening, requires or prolongs hospitalization, results in persistent or significant disability or incapacity, causes a congenital anomaly or birth defect, or is otherwise considered medically significant by the investigator.

All AEs and SAEs will be recorded throughout the intervention and follow-up periods, including the event description, onset and resolution dates, severity, seriousness, management, outcome, and relationship to the study intervention. The severity of AEs will be classified as mild, moderate, or severe. The relatedness of each AE to the study intervention will be classified as unrelated, unlikely related, possibly related, probably related, or definitely related.

Expected acupuncture-related AEs include local pain, bleeding or hematoma, infection, dizziness or syncope, retained or broken needles, and pneumothorax. Other unexpected AEs will also be documented. Antiplatelet and anticoagulant therapy will be permitted when clinically indicated and will not be routinely

discontinued for acupuncture. Precautions will include gentle needling, careful needle insertion and removal, and observation for bleeding and pneumothorax after treatment.

At each treatment session and scheduled follow-up visit, participants will be actively asked about AEs, and spontaneous reports will be recorded whenever they occur, including a description of the event, the onset and end times, severity, frequency, and its relationship with the study conduct and acupuncture, the actions taken and the final prognosis (resolved, improved, with sequelae and death) in the CRFs. We will follow up the adverse events that lead to withdrawal or persist at the end of the study until an outcome occurs.

Serious adverse events need to be recorded in the CRF and reported to the principal investigator (YW) within 24 hours. All serious adverse events that remain unresolved by the end of the study, or at the time a participant discontinues study participation, must be followed until the event resolves, stabilizes or until a final outcome occurs.

Acupuncture will be temporarily interrupted or discontinued in participants who develop an acupuncture-related SAE, disease deterioration requiring treatment that precludes acupuncture, or when continuation of acupuncture is considered unsafe by the treating acupuncturist.

6. ePRO–EDC Data Integration

Participants will access the ePRO system through the designated WeChat Official Account to complete weekly CCQ assessments. The WeChat Official Account serves as the access interface to the ePRO system, through which participant information and CCQ responses are collected and automatically synchronized with the integrated EDC system. The integrated ePRO–EDC system supports study data collection, automated alert generation, and study data management, while the WeChat-based interface facilitates alert delivery and communication between participants and research personnel.

Relevant study data, including CCQ assessments, alert records, clinical assessments, and study outcomes, will be managed using pseudonymized participant study IDs. The ePRO system will retain the necessary linkage between study IDs and participant identifiers to support participant identification, longitudinal monitoring, and alert delivery, whereas directly identifiable information will not be routinely included in the EDC study dataset. Access to the WeChat-based ePRO interface, integrated ePRO–EDC system, and participant-identification information will be restricted to authorized study personnel. Data transmission and storage will be protected by appropriate security and encryption measures, with routine backup and recovery procedures implemented. Study data will be retained for the period required for study conduct and applicable regulatory requirements.

7. Handling of Missing Data

Missing outcome data will be imputed under the missing at random assumption using the multiple imputation chained equation (MICE) algorithm (function mice in R). Predictive mean matching, in which imputed values are sampled only from the observed values, will be used [1]. A total of 50 imputed datasets will be generated [2]. The random seed will be 128. Variables used to impute missing data will include all outcomes at all follow-up time points, trial arm status, center, age, history of acute exacerbations/prior hospitalizations, smoking status and coronary artery disease. Each imputed dataset will be analyzed with the prespecified baseline-adjusted ANCOVA models, and effect estimates and standard errors will be combined using Rubin's rules. As a sensitivity analysis for departures from the MAR assumption, multiple imputation will first be performed under MAR using the prespecified MICE procedure, after which the imputed values will be shifted toward worse outcomes by 0.2 and 0.5 standard deviations (SDs) of the corresponding outcome [3].

To account for death-related missingness, we will adopt a 'composite-outcome' strategy to incorporate death in the outcome and assign a prespecified value to participants who died [3]. In this study, we will assign 10 to the Borg Dyspnea Scale (i.e., the maximal breathlessness), 100 to SGRQ (i.e., worst possible health status), and 0 to FEV_1% predicted (i.e., the worst possible function). Under the 'composite-outcome' strategy, the estimand is the "the mean difference between the intervention and control groups in the change from baseline to 12 months in the Borg Dyspnea Scale, SGRQ, or FEV1% predicted, with participants who died assigned a score of 10, 100, or 0, respectively". For the 'composite-outcome' strategy, the 'impute then delete' approach will be used to handle missing outcome data [3]. Multiple imputation will first be performed for missing scores at all scheduled follow-up time points. After completion of the multiple imputation procedure, the imputed values after death will be deleted and replaced with the prespecified assigned scores (see above) to form the composite outcome.

The 'while-alive' strategy will be used as a sensitivity analysis for handling outcomes truncated by death [4]. Under the 'while-alive' strategy, the estimand is the "the mean difference between the intervention and control groups in the change from baseline to 12 months in the Borg Dyspnea Scale, SGRQ, or FEV1% predicted, at the last point participants were alive, up to 12 months". For the 'while-alive' strategy, the 'impute then delete' approach will be used to handle missing outcome data [3]. Multiple imputation will first be performed for missing outcome scores at all scheduled follow-up time points, including time points after death using the prespecified imputation model (see above). After multiple imputation, imputed outcome values at time points after death will be discarded. For participants who die before 12 months, the outcome will be defined as the latest observed outcome score before death or, if the outcome at that assessment is missing, the corresponding imputed outcome score. For participants who remain alive at 12 months, the 12-month outcome score will be used.

References

1. White, I.R.; Royston, P.; Wood, A.M. Multiple imputation using chained equations: Issues and guidance for practice. Stat. Med. 2011, 30, 377-399, doi:10.1002/sim.4067.
2. Austin, P.C.; White, I.R.; Lee, D.S.; van Buuren, S. Missing Data in Clinical Research: A Tutorial on Multiple Imputation. Can. J. Cardiol. 2021, 37, 1322-1331, doi:10.1016/j.cjca.2020.11.010.
3. Cro S, Morris TP, Kenward MG, Carpenter JR. Sensitivity analysis for clinical trials with missing continuous outcome data using controlled multiple imputation: A practical guide. Statistics in Medicine. 2020;39(21):2815–2842. doi:10.1002/sim.8569.
4. Pham, T.M.; Kahan, B.C.; Lopes, A.; Rashid, M.; Hoskin, P.J.; White, I.R. Estimating treatment effects in trials with outcome data truncated by death: A case study on aligning estimators with estimands. Clin. Trials 2025, 22, 676-686, doi:10.1177/17407745251360645.
