# Supplementary material for: An Integrated Electronic Patient-Reported Outcome-Guided COPD Management Strategy Incorporating Adjunctive Acupuncture: Study Protocol for a Randomized Controlled Trial
Source: Healthcare (Basel). 2026 Oct 19;14(20):3509. doi: 10.3390/healthcare14203509 (PMC13704764; doi:10.3390/healthcare14203509)
Supplement: Supplementary file 1 [file healthcare-14-03509-s001.zip › Supplementary File S2.pdf]

Supplementary File S2. Details of the Administration of Acupuncture

Patients assigned to the intervention group will receive 12 sessions of acupuncture after randomization, scheduled at 3 sessions per week for 4 consecutive weeks. Each treatment session must be scheduled with a permitted interval window of 2–3 days between consecutive sessions. No co-interventions (e.g., moxibustion, cupping, herbal medicine, exercise, or lifestyle advice) will be administered alongside acupuncture.

This trial will adopt a fixed-formula traditional Chinese medicine acupuncture approach with a predefined acupoint prescription; no individualized point adjustment or supplementary additional acupoints are permitted. The selected acupoints were chosen based on our previous study, including Zhongfu (LU1), Dingchuan (EX-B1), Feishu (BL13), Pishu (BL20), Shenshu (BL23), Zusanli (ST36), and Fenglong (ST40). The rationale for this acupoint selection is as follows: Zhongfu (LU1) and Feishu (BL13) are selected to regulate lung qi; Dingchuan (EX-B1) is used for relieving dyspnea; Pishu (BL20) serves to tonify the spleen; Shenshu (BL23) reinforces the kidney; Zusanli (ST36) tonifies the spleen-stomach; and Fenglong (ST40) eliminates phlegm. All seven acupoints are needled bilaterally, resulting in 14 needle insertions in total for each treatment session. No other acupoints will be allowed. The location of acupoints and the direction and depth of needle insertion are shown in eTable 1 and eFigure 1 [1].

Licensed acupuncturists who have at least 3 years of experience with acupuncture will administer the treatment. We will use disposable sterile Huatuo acupuncture needles (0.25 mm × 40 mm and 0.25 mm × 25 mm) and follow the protocol below:

1) Place the patient in a seated position.

2) Disinfect the targeted acupoint areas with 75% alcohol using sterile tweezers and a sterile cotton ball.

3) Insert needles into the selected acupoints (eFigure 1).

4) Conduct needle manipulation to elicit de qi, which refers to a composite of sensations such as soreness, numbness, distension, heaviness, and related feelings [2].

5) Retain the needles for 30 min and manipulate the needle every 10 min for 15 seconds.

6) Acupuncturists must strictly control the direction and depth for the back and chest acupoints (i.e., Zhongfu (LU1), Dingchuan (EX-B1), and Feishu (BL13)). Excessive lifting–thrusting or large twisting manipulations at these acupoints are not permitted.

Table S1. The location of acupoints and the direction and depth of needle insertion.

Acupoint	Area	Location	Needle insertion	Assigned needle length
Zhongfu (LU1)	Chest region	On the upper lateral chest, 6 cun from the middle of the chest, level in the first intercostal space.	Angled outward, 0.5–0.8 cun	0.25 × 25 mm
Dingchuan (EX-B1)	Cervical region	0.5 cun horizontally next to the depression below the spinous process of the 7th cervical spine.	Vertically or slightly inward, 0.5–0.8 cun	0.25 × 25 mm
Feishu (BL13)	Dorsal region	1.5 cun horizontally next to the depression below the spinous process of the 3rd thoracic vertebra.	Lateral inclination of 45 degrees, 0.5–0.8 cun	0.25 × 25 mm
Pishu (BL20)	Dorsal region	1.5 cun horizontally next to the depression below the spinous process of the 11th thoracic vertebra.	Vertically, 0.5–0.8 cun	0.25 × 25 mm
Shenshu (BL23)	Lumbar region	1.5 cun horizontally next to the depression below the spinous process of the 2nd lumbar spine.	Vertically, 0.5–1 cun	0.25 × 40 mm
Zusanli (ST36)	Lateral calf region	3 cun below the Dubi (ST35) on the line between Dubi (ST35) and Jiexi (ST41). Note: ST35 is located in the depression on the	Vertically, 1–2 cun	0.25 × 40 mm

		lateral side of the patellar ligament in the anterior region of the knee; ST41 is located in the depression in front of the ankle joint between the extensor pollicis longus tendon and the extensor digitorum longus tendon.		
Fenglong (ST40)	Lateral calf region	8 cun above the tip of the lateral malleolus, lateral margin of the tibialis anterior muscle, 1 cun outside the Tiaokou (ST38). Note: ST38 is 8 cun below ST35 on the lateral side of the calf and on the line between ST35 and ST41.	Vertically,<br>1–1.5 cun	0.25 × 40 mm

Note: Cun is the unit of length. The width of a person's thumb at the knuckle is 1 cun. We refer to GB/T 12346-2021 for all the nomenclature and location of meridian acupoints [1].

Figure S1. Schematic diagram of acupuncture points.

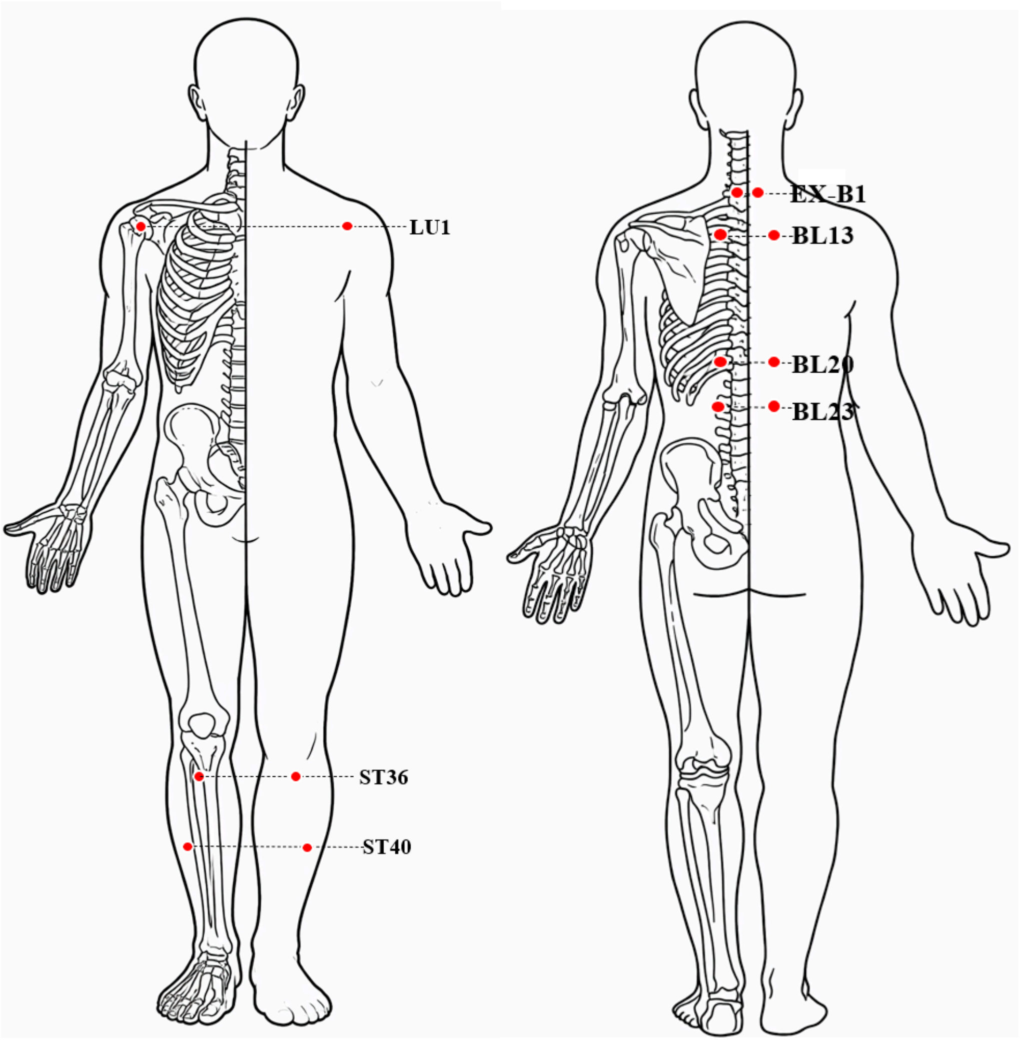

Table S2. STRICTA 2010 checklist for acupuncture intervention reporting.

Item	Detail
**1. Acupuncture rationale**	1a) This trial will adopt a fixed formula traditional Chinese medicine acupuncture approach with a predefined acupoint prescription.
	1b) The rationale for this acupoint selection is as follows: Zhongfu (LU1) and Feishu (BL13) are selected to regulate lung qi; Dingchuan (EX B1) is used for relieving dyspnea; Pishu (BL20) serves to tonify the spleen; Shenshu (BL23) reinforces the kidney; Zusanli (ST36) tonifies the spleen stomach; and Fenglong (ST40) eliminates phlegm.
**2. Details of needling**	2a) 14 needle insertions in total for each treatment session. No other acupoints will be allowed.
	2b) All seven acupoints are needled bilaterally, including Zhongfu (LU1), Dingchuan (EX-B1), Feishu (BL13), Pishu (BL20), Shenshu (BL23), Zusanli (ST36), and Fenglong (ST40).
	2c) Depth of insertion is shown in eTable 1.
	2d) Conduct needle manipulation to elicit de qi, which refers to a composite of sensations such as soreness, numbness, distension, heaviness, and related feelings.
	2e) Manipulate needle every 10 min for 15 seconds.
	2f) Retain the needles for 30 min.
	2g) We will use disposable sterile acupuncture needles of Huatuo brand (0.25 × 40 mm and 0.25 × 25 mm).
**3. Treatment regimen**	3a) Patients assigned to the intervention group will receive 12 sessions of acupuncture after randomization.
	3b) 3 sessions per week for 4 consecutive weeks. Each treatment session must be scheduled with a permitted interval window of 2–3 days between consecutive sessions.
**4. Other components of treatment**	4a) No co-interventions (e.g., moxibustion, cupping, herbal medicine, exercise, or lifestyle advice) will be administered alongside acupuncture.
	4b) Licensed acupuncturists who have at least 3 years of experience with acupuncture will administer the treatment. We will use disposable sterile Huatuo acupuncture needles (0.25

	mm × 40 mm and 0.25 mm × 25 mm) and follow the protocol below: 1) Place the patient in a seated position. 2) Disinfect the targeted acupoint areas with 75% alcohol using sterile tweezers and a sterile cotton ball. 3) Insert needles into the selected acupoints (eFigure 1); 4) conduct needle manipulation to elicit de qi, which refers to a composite of sensations such as soreness, numbness, distension, heaviness, and related feelings. 5) Retain the needles for 30 min and manipulate the needle every 10 min for 15 seconds. 6) Acupuncturists must strictly control the direction and depth for the back and chest acupoints (i.e., Zhongfu (LU1), Dingchuan (EX-B1), and Feishu (BL13)). Excessive lifting–thrusting or large twisting manipulations at these acupoints are not permitted.
**5. Practitioner background**	5) Licensed acupuncturists who have at least 3 years of experience with acupuncture will administer the treatment.

Reference

1. *GB/T 12346-2021*; Nomenclature and Location of Meridian Points. State Administration for Market Regulation, Standardization Administration of China: Beijing, China, **2021**
2. Zhou, K.; Fang, J.; Wang, X.; Wang, Y.; Hong, Y.; Liu, J.; Wang, L.; Xue, C.; Wang, P.; Liu, B.; et al. Characterization of De Qi with Electroacupuncture at Acupoints with Different Properties. *The Journal of Alternative and Complementary Medicine* **2011**, *17*, 1007-1013, doi:10.1089/acm.2010.0652.
